# diffReps: Detecting Differential Chromatin Modification Sites from ChIP-seq Data with Biological Replicates

**DOI:** 10.1371/journal.pone.0065598

**Published:** 2013-06-10

**Authors:** Li Shen, Ning-Yi Shao, Xiaochuan Liu, Ian Maze, Jian Feng, Eric J. Nestler

**Affiliations:** 1 Fishberg Department of Neuroscience and Friedman Brain Institute, Icahn School of Medicine at Mount Sinai, New York, New York, United States of America; 2 Laboratory of Chromatin Biology and Epigenetics, The Rockefeller University, New York, New York, United States of America; Università degli Studi di Milano, Italy

## Abstract

ChIP-seq is increasingly being used for genome-wide profiling of histone modification marks. It is of particular importance to compare ChIP-seq data of two different conditions, such as disease vs. control, and identify regions that show differences in ChIP enrichment. We have developed a powerful and easy to use program, called diffReps, to detect those differential sites from ChIP-seq data, with or without biological replicates. In addition, we have developed two useful tools for ChIP-seq analysis in the diffReps package: one for the annotation of the differential sites and the other for finding chromatin modification “hotspots”. diffReps is developed in PERL programming language and runs on all platforms as a command line script. We tested diffReps on two different datasets. One is the comparison of H3K4me3 between two human cell lines from the ENCODE project. The other is the comparison of H3K9me3 in a discrete region of mouse brain between cocaine- and saline-treated conditions. The results indicated that diffReps is a highly sensitive program in detecting differential sites from ChIP-seq data.

## Introduction

ChIP-seq (Chromatin immunoprecipitation followed by deep sequencing) is the state-of-the-art technology for genome-wide profiling of protein-DNA interaction sites [1]. Using an antibody against a protein of interest, crosslinked DNA-protein complexes are enriched and selected, then the ChIP-DNA fragments are sequenced, and protein-DNA interaction sites can be profiled at a whole-genome scale. This has provided us with unprecedented details [2] of protein-DNA interactions at genes, as well as intergenic regions, in comparison with its predecessor, ChIP-chip. Previous work had largely been devoted to the identification of the locations of these interaction sites [3], i.e., peak calling. However, less attention has been paid to the discrepancies of the intensity of these interactions under different conditions. As differences of histone modifications can often associate with functional consequences, it has become an urgent task to identify the differential sites from ChIP-seq data.

Some groups have already focused on this task and a few tools have been developed to date [4], [5], [6]. Some of them rely on peak calling programs to identify the interaction sites first, and then perform differential analysis based on peak intensity comparisons. However, peak calling for chromatin modification marks is still poorly established, especially for the long and diffusive marks. An example is H3K36me3, a histone mark for transcriptional elongation, which often spans the whole gene body. Different peak calling programs often give very different results, making the choice of peak calling algorithm difficult. In addition, small changes are often observed within a large peak, especially from an *in vivo* study, making the peak-calling-dependent approaches less powerful. Those small changes are not trivial and can often associate with biological functions. For example, many histone marks have been implicated in the regulation of pre-mRNA alternative splicing [7]. Indeed, a large portion of differential sites are observed on or around exons. Another limitation of some of the existing methods is that they do not take biological replicates into account. Using biological replicates is crucial for an *in vivo* study where the variation, derived from biology or experimental variability, is typically large. It has been recognized that at least 2–3 replicates are necessary for *in vivo* sequencing analyses [1].

To address these challenges, we have developed diffReps, a program to detect differential sites from two comparison groups of ChIP-seq samples. diffReps is independent of any peak calling program and provides several statistical tests to take advantage of the biological replicates. We have also taken the task of identifying regions where the differential sites occur significantly more often than chance, or the so-called chromatin modification hotspots. Applying diffReps to study the differential sites of H3K4me3 between hESC (human embryonic stem cells) and K562 (leukemia cells) from ENCODE, we found a large number of differential sites to associate strongly with gene expression changes and alternative splicing. We also applied diffReps to our previously published ChIP-seq data of chronic cocaine-regulated H3K9me3 in mouse nucleus accumbens (NAc) [8] and found numerous hotspots which may associate with altered nervous system functions.

## Results

### Model Description

diffReps is designed as a PERL program that can analyze an entire ChIP-seq dataset using only one command. It uses a sliding window to scan the genome and identifies the ones that show read count differences. A sliding window is defined as a fix-sized genomic region where the reads falling into this region can be counted. The sliding window moves along the genome in a fixed step size that is typically a fraction of the window size. The reason to use these partially overlapping windows is to increase the resolution of differential site detection. A user can specify the window and step size as program arguments. In this paper, we use window size of 1Kb and step size of 100bp across the board. To ensure fair comparison, the same window size is used for all the other comparing methods. This sliding window strategy allows diffReps to be independent of any peak calling program. After the detections are done, diffReps merges the overlapping windows that pass a predefined statistical cutoff to define a so called differential site. The statistical significance for each differential site is then re-evaluated. It also records the sliding window that shows the most significance among each differential site as part of its report. Finally, multiple testing correction is performed on the differential sites for statistical stringency. diffReps works with reasonable requirements of memory and CPU power: it typically finishes running within 3 hr and uses <4GB RAM with a mammalian genome size dataset on a regular workstation (see Text S1 for details). Once the differential sites are found, diffReps automatically classifies each of them into one of the following categories (see Design and Implementation for more details): Proximal Promoter, Promoter1k, Promoter3k and Genebody. If a neighboring gene cannot be found, diffReps attempts to associate a differential site with one of the following heterochromatic regions: Genedesert, Pericentromere and Subtelomere. If none of the above can be assigned, the differential site is classified as “Other Intergenic”. So far, we have compiled genomic annotations for three model species: mouse (mm9), human (hg19) and rat (rn4), with more to be added in the future. Users can also choose between two database flavors for gene annotation: RefSeq and Ensembl.

The number of differential sites in a local region can be approximated by a Poisson distribution if all differential sites were allocated randomly on the genome. However, differential sites are often found to be spatially clustered, forming so called chromatin modification hotspots. These hotspots represent the heavily regulated parts of a genome, which may be functionally important under certain biological conditions. diffReps finds the hotspots by first building null models (both local and global) on differential site density and then looking for regions that violate the null models with statistical significance using a “greedy search” algorithm. In addition, when more than one lists of different histone marks are presented to diffReps, they are first combined and sorted according to the differential sites’ genomic coordinates and then treated like a single list. Our program can recognize the histone mark type for each differential site and report the histone marks that are involved in each hotspot. This way, a user can easily identify the interactions between different histone marks in the hotspots and use this information for classification purposes later.

### Identifying Cell-specific Chromatin Modifications

We applied diffReps to analyze the differential sites of H3K4me3 between hESC and K562 from the ENCODE project [9]. This dataset (see Text S1 for more details) contains two biological replicates for each condition and the number of uniquely mapped reads ranges between 7 and 16 million (Table 1). We used diffReps with default parameter values except that the “–nsd” option (see Design and Implementation) is set to “sharp” because H3K4me3 tends to generate sharp peaks. The hESC was used as the control group while the K562 was used as the treated group. In addition, two input samples are also available in each cell line from the same project (see Text S1 for more details). We mixed one input from hESC with another input from K562 and created two groups of input samples. We used these data to perform mock comparisons to estimate the number of false positives given by a differential analysis method.

**Table 1:** Summary of the two benchmark datasets.

	*In vivo* mouse brain		Human cell culture	
**Mark**	H3K9me3		H3K4me3	
**Genome**	Mm9		Hg19	
**Condition Name**	**Saline**	**Cocaine**	**hESC**	**K562**
	**#Mapped reads**			
**Replicate 1**	13132622	14324424	7232113	9593249
**Replicate 2**	14399342	12697989	14188117	16311376
**Replicate 3**	15092182			

The numbers reported are uniquely mapped short reads.

First, we set out to determine the sensitivity and specificity (using mock data) of a number of different methods, including diffReps (negative binomial test), diffReps (G-test on pooled replicates), DESeq [10], edgeR [11] and ChIPDiff [6]. Both DESeq and edgeR are popular R packages that are widely used in differential expression analysis for RNA-seq. They use the negative binomial distribution to estimate the over-dispersion among replicates and further stabilize it using neighboring genes. They can also be applied to ChIP-seq data by splitting the genome into non-overlapping windows (see Text S1 for more details). ChIPDiff is an approach based on hidden Markov models (HMM) to detect differential sites. For completion, we also added a peak-calling based approach by first calling H3K4me3 peaks using CCAT [12], finding the union of peaks from the two cell lines and then identifying differential peaks using DESeq (referred in the following as CCAT+DESeq). This way we can compare the peak-calling based approach with window based methods.

**Figure 1:**
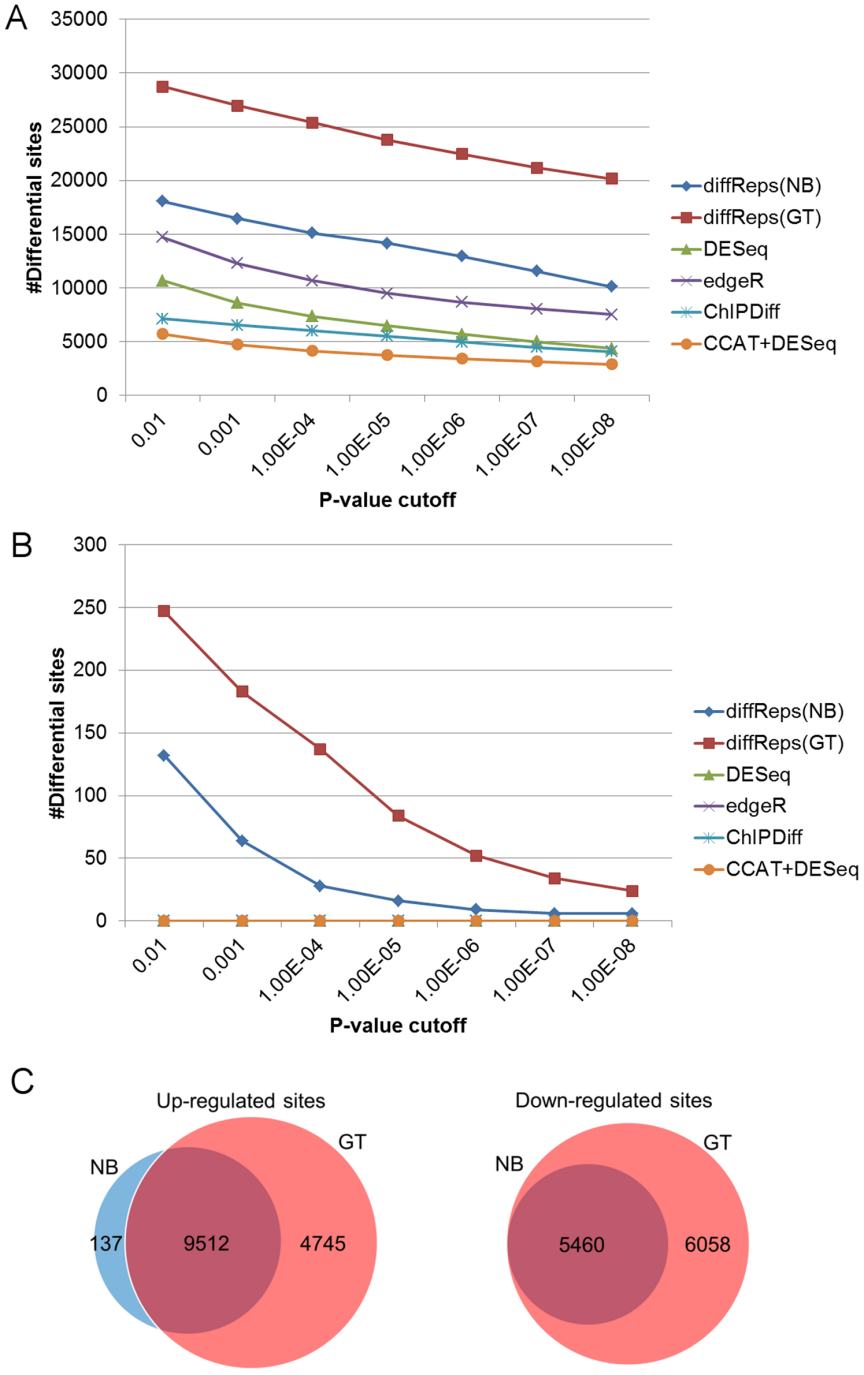
The number of differential sites found by different methods as p-value cutoff varies from 1E-2 to 1E-8 on the ENCODE H3K4me3 ChIP-seq data. NB = negative binomial test. GT = G-test. X-axis represents the different p-value cutoffs. Y-axis represents the number of differential sites. (A) Sensitivity curves based on the H3K4me3 ChIP data; (B) Specificity curves based on the DNA input mock data; (C) Venn diagrams of NB vs. GT using diffReps based on its default cutoff (*p<1E-4*).

Each method is run repeatedly with its nominal p-value chosen from 1E-2 to 1E-8 (See Figs. S3 & S4 for additional p-values). Fig. 1A shows that diffReps is the most sensitive method among all the methods compared, followed by edgeR, DESeq, ChIPDiff and, lastly, CCAT+DESeq. At each cutoff, diffReps (negative binomial test) typically detects a few thousands more differential sites than the secondly ranked method, edgeR. It should be noticed that G-test predicts ∼10,000 more sites than negative binomial test on average. However, G-test also generates ∼4–5 times more false positives than negative binomial test when using a p-value cutoff of 1E-4 or more stringent (Fig. 1B). Because G-test simply considers the ratio of read count between two comparison groups and ignores the variation within a group, some loss of specificity is expected. What is more, the negative binomial test result is almost a subset of the G-test result as shown by Venn diagrams (Fig. 1C). This makes G-test a viable alternative to negative binomial test if specificity is less of a concern. Although diffReps seems to produce more false positives than the other methods on the mock data (Fig. 1B), we observed an exponential decrease of false positives when the significance cutoff increases. At diffReps’ default cutoff (*p<1E-4*), the empirically estimated false discovery rate (FDR) is 28/15,109 = 0.2% for negative binomial test and 137/25,369 = 0.5% for G-test.

**Figure 2:**
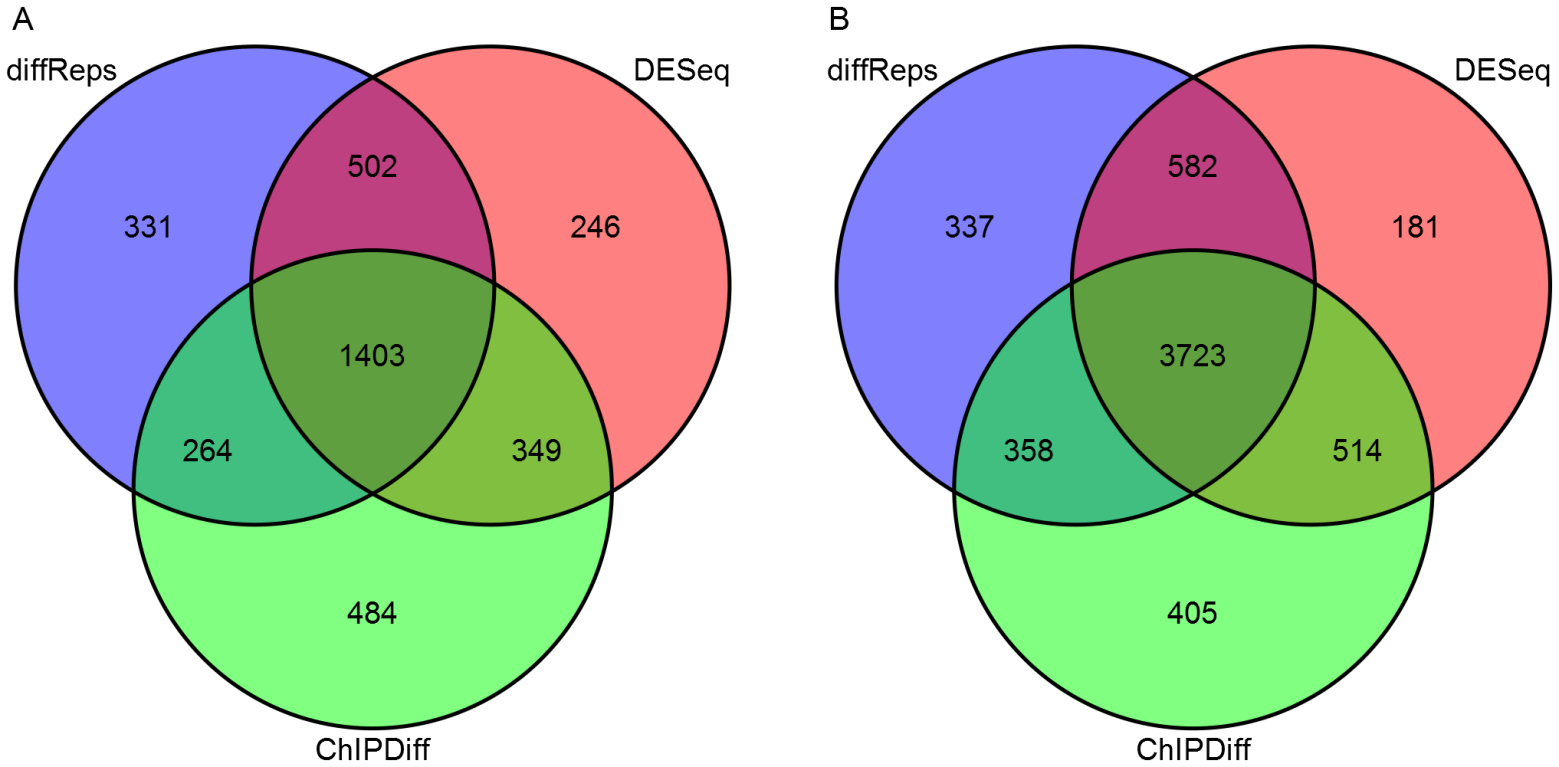
Overlap of the top differential site lists of three methods: diffReps (negative binomial test), DESeq and ChIPDiff on H3K4me3 comparing K562 and hESC. (A) Top 2,500; (B) Top 5,000.

Another experiment was performed to determine the consistency among various methods in ranking differential sites by statistical significance. diffReps (negative binomial test) was compared against two other approaches on the top 2,500 and 5,000 sites using Venn diagrams. Fig. 2 shows that diffReps shows very nice consistency with DESeq and ChIPDiff: 87% (2,500) and 93% (5,000) of the diffReps top sites overlap with another method. In contrast, ChIPDiff shows slightly more disparity than the other methods: 19% (2,500) and 8% (5,000) of its top sites are unique. We performed the same analysis between diffReps (negative binomial test), edgeR and CCAT+DESeq. diffReps shows very nice consistency with edgeR (Fig. S1): 74% (2,500) and 82% (5,000) of the top sites are overlapped. However, CCAT+DESeq largely disagrees with the other methods: 78% (2,500) and 80% (5,000) of its top sites are unique.

**Figure 3:**
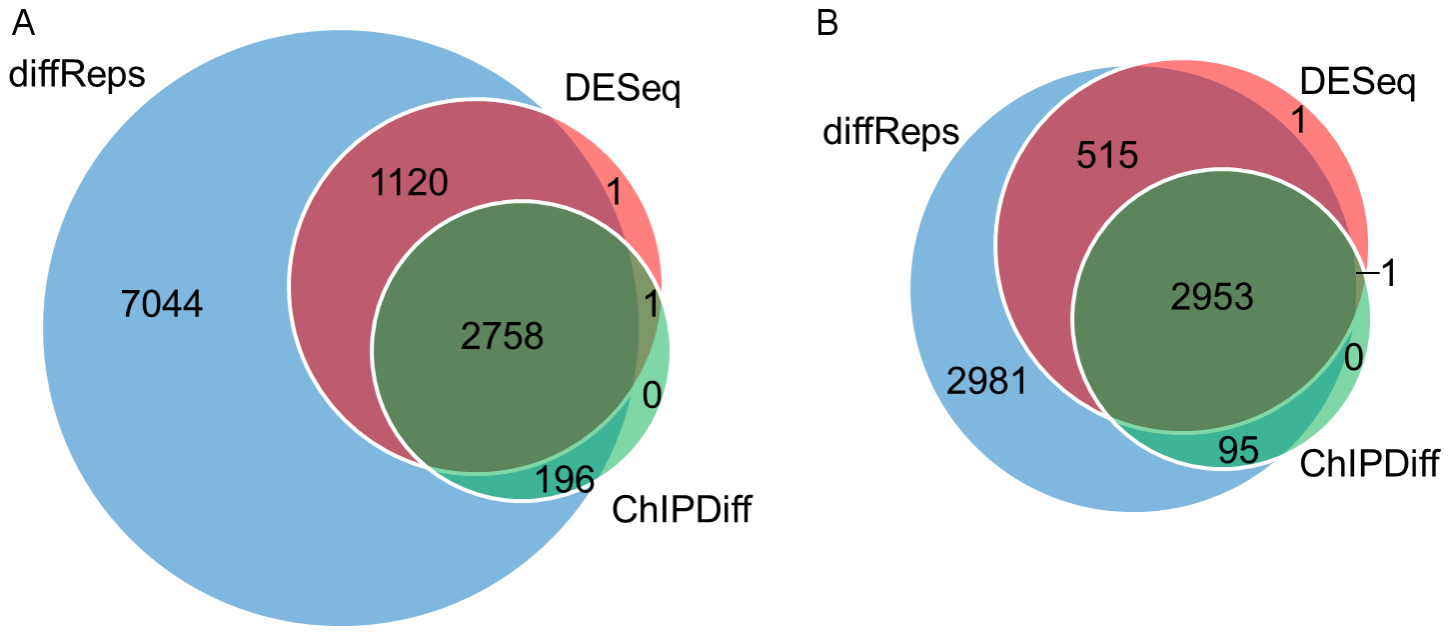
Venn diagrams of the differential sites of diffReps (negative binomial test), ChIPDiff and DESeq on H3K4me3 comparing K562 and hESC. Because a differential site from one method may overlap with two or more sites from another method, a priority is set for the number to be reported at the overlapped regions: ChIPDiff>DESeq>diffReps. (A) Increased sites; (B) Decreased sites.

To further study the differential sites identified by diffReps (negative binomial test), we compared it with ChIPDiff and DESeq at its default significance cutoff (*p<1E-4*). In total, diffReps found 9,649 increased sites and 5,460 decreased sites in K562 vs. hESC. ChIPDiff detected 2,955 increased and 3049 decreased sites, while DESeq detected 3,880 increased and 3,470 decreased sites. Venn diagram shows that almost all of the differential sites found by ChIPDiff and DESeq are also detected by diffReps (Fig. 3).

H3K4me3 is known to be associated with transcriptional activation [13], [14] and may also relate to alternative splicing [15]. To study the functional relevance of the differential sites, we analyzed the transcriptomes of hESC and K562 using RNA-seq. Cufflinks [16] was used to identify differential transcriptomic events (FDR<5%) between the two cell lines [17]. We separated all differential sites detected by the three approaches (diffReps, ChIPDiff and DESeq) into two categories: “diffReps-specific” and “Overlap” (i.e., detected by both diffReps and another method). To correlate the differential sites with gene expression, we further classified them into “Promoter” (upstream 1Kb to downstream 1Kb of TSS) and “Genebody” (downstream 1Kb of TSS to TES), and ignored the ones that are mapped to intergenic regions. Considering the direction of change, we defined four groups in total: “Promoter Up”, “Promoter Down”, “Genebody Up” and “Genebody Down”.

**Figure 4:**
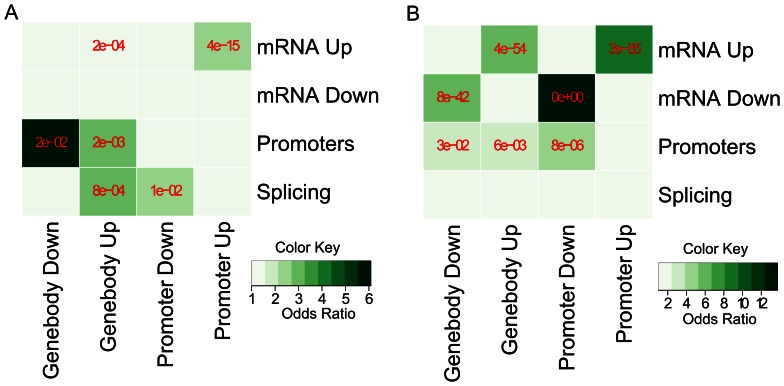
Heatmaps of correlation between chromatin modification differential sites and differential transcriptomic events on the ENCODE data. mRNA Up, mRNA Down: overall gene expression change. Promoters: alternative promoter usage. Splicing: alternative splicing. The darkness of the color indicates the odds ratio (i.e. observation/expectation) of enrichments. The *p-values* of enrichments are determined by Fisher’s exact test and are labeled in red color. (A) diffReps-specific: differential sites detected only by diffReps; (B) Overlap: differential sites detected by both diffReps and another method.

**Figure 5:**
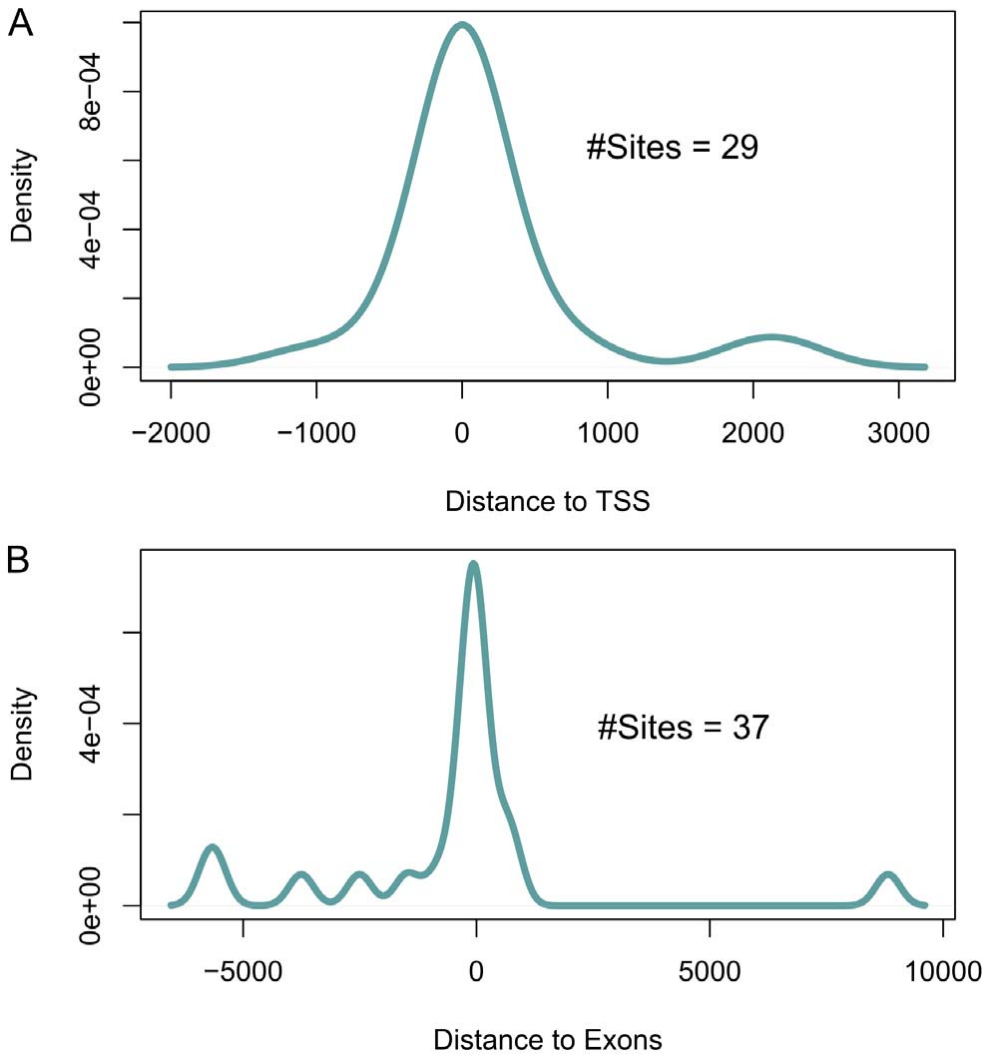
Distance density plot of diffReps-specific sites that are related with alternative promoter usage and alternative splicing. The distance is determined by first looking for the closest TSS or alternative exon and then calculating the number of basepairs between the boundaries of a site and a feature. (A) Distance to TSS; (B) Distance to exon.

In the overlap category, the differential sites show very significant and positive correlation with the overall gene expression change (Fig. 4B and Table S3), validating the function of H3K4me3 as an activation mark. These differential sites also show some correlation with alternative promoter usage but no correlation with alternative splicing (Fig. 4B and Table S3). In the diffReps-specific category, only increased gene expression is correlated with “Promoter Up” and “Genebody Up” (Fig. 4A and Table S3). Interestingly, alternative splicing shows correlation with “Genebody Up” and “Promoter Down”, while alternative promoter shows correlation with “Genebody Down” and “Genebody Up” (Fig. 4A and Table S3). There are 61 and 69 genes that show alternative promoter usage and alternative splicing on the genome, while 20 and 14 of them contain at least one diffReps-specific site. In total, 29 and 37 diffReps-specific sites are located on those genes with alternative promoter usage and alternative splicing, respectively. We identified those sites’ closest TSS or alternative exon (i.e., variant exon and exons with alternative boundaries) and plotted the distribution of the distance between a site and its closest feature (Fig. 5). Clearly, there is a sharp peak centered on TSS or exon in the density plots, indicating spatial proximity of the differential sites to those features. To investigate whether this distribution is specific to the sites that are related with alternative splicing/promoter, we drew the same plots for all 10,025 diffReps-specific sites (Fig. S2). We observed the same distribution pattern. Indeed, 8,410 (or 84%) of the sites are within 1Kb of TSSs or alternative exons, corresponding to 7,120 unique genes. 638 of these genes show transcriptional changes (i.e. whole gene expression, alternative promoter or alternative splicing) as defined by Cufflinks (FDR<5%).

As a negative control, we performed the same correlation analysis between the differential sites from the mock data and the transcriptional changes. In total, diffReps found 28 (negative binomial test) and 137 (G-test) differential sites of which only 3 and 12 can be mapped to promoter or genebody, respectively. None of these differential sites overlaps with the transcriptional changes.

**Figure 6:**
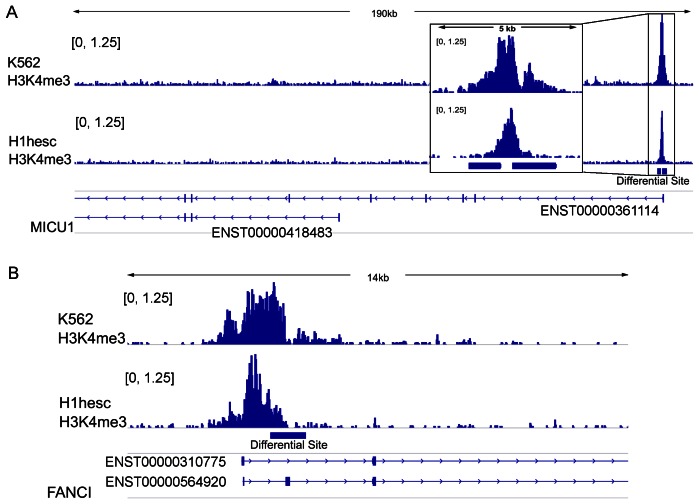
IGV [34] genome browser screenshots of two example genes that contain alternative promoter usage and alternative splicing events. The top two tracks are normalized genomic coverage of H3K4me3 in K562 and hESC cell lines. They are overlaid by diffReps-specific sites shown as solid bars. The bottom track is the gene model with two representative isoforms. (A) Gene MICU1 contains alternative promoter usage between two isoforms. The inset is a magnified figure of the differential sites at TSS; (B) Gene FANCI contains alternative splicing with a variant exon being preferentially included in K562 vs. hESC.



Presumably the overlap category contains differential sites that are most significant and represent major H3K4me3 differential sites. It is not surprising to find them to correlate with gene expression change. While the diffReps-specific sites represent minor H3K4me3 differential sites. The correlation between diffReps-specific sites and alternative splicing (as well as gene expression) indicates that many functionally important differential sites may be missed by other methods. This is further illustrated by two examples (Fig. 6). MICU1, a gene that encodes a mitochondrial calcium uptake protein, contains two isoforms with alternative TSSs: one TSS is about 100Kb downstream of the other. ENST00000361114 is a major isoform that shows 7.4 fold increased expression in K562 (RPKM = 37.2) compared to hESC (RPKM = 5.0). While ENST00000418483 is a minor isoform that shows the opposite direction of change (hESC RPKM = 2.5; K562 RPKM = 0). diffReps found two up-regulated H3K4me3 sites: one (fold change = 2.5; p-value = 1.3E-5) is ∼500bp upstream and the other (fold change = 2.1; p-value = 6.8E-7) is ∼1,200bp downstream of the TSS of ENST00000361114 (Fig. 6A). This supports the role of H3K4me3 as a positive regulator of alternative promoter usage. FANCI is a gene that belongs to the Fanconi anemia complementation group which is related to DNA repair. This gene’s expression shows a 6.4 fold increase from hESC (RPKM = 14.3) to K562 (RPKM = 91.4). However, the gene’s 18 isoforms show variable degrees of transcriptional change which is probably regulated by the splicing machinery in a very delicate way. Cufflinks can detect these splicing changes by modeling the isoform expression distributions for the two comparison groups [16] and report alternative splicing events. Here the gene is determined by Cufflinks to contain alternative splicing (FDR<0.1%). To evaluate the degree of splicing vs. overall transcriptional output for each isoform, a splicing index can be calculated as:

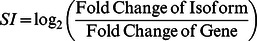



so that a negative *SI* means the isoform changes to a lesser degree than the other isoforms; while a positive *SI* means the isoform changes to a greater degree than the other isoforms. A major isoform, ENST00000310775, shows a 5.0 fold increase from hESC (RPKM = 5.6) to K562 (RPKM = 28.2), while a minor isoform, ENST00000564920, shows a 34.0 fold increase from hESC (RPKM = 0.1) to K562 (RPKM = 3.4). The *SI* is calculated for the major and minor isoforms to be −0.4 and 2.4, respectively. diffReps found an up-regulated site (fold change = 2.5; p-value = 3.3E-6) which overlaps a variant exon of the minor isoform (Fig. 6B). This seems to suggest a positive role for H3K4me3 in the inclusion of the variant exon in K562 cells.

It should be noticed that in Fig. 6B, the differential site overlaps with an H3K4me3 peak that sits on the TSS of FANCI. H3K4me3 is a histone mark that strongly associates with promoters (or alternative promoters). In our analysis, we also found most differential sites to be located near TSS. However, an H3K4me3 peak often spans several Kb, raising the possibility for it to affect the splicing procedures of the downstream overlapping exons. Previously, people have reported the histone mark’s association with splicing [15]. Here we have shown some statistical association between the mark’s differential sites that extend into genebody and splicing. This phenomenon is further demonstrated by an additional example in Fig. S7.

### Applying diffReps to Study a Repressive Mark in Mouse Brain

We have previously published a ChIP-seq study [8] of H3K9me3 in nucleus accumbens of mouse treated with chronic cocaine or saline injections. This dataset (Table 1) contains two groups: cocaine and saline. The cocaine group has two replicates and the saline group has three replicates. Each replicate has ∼12–15 million uniquely mapped reads. This dataset also contains six DNA input samples. We separated them into two groups with three samples in each group and created a mock dataset. This dataset represents an *in vivo* study where the signal-to-noise ratio is presumably much lower than that of cultured cells. We used diffReps with default parameter values and the “–nsd” option is set to “broad” because H3K9me3 tends to generate broad peaks.

**Figure 7:**
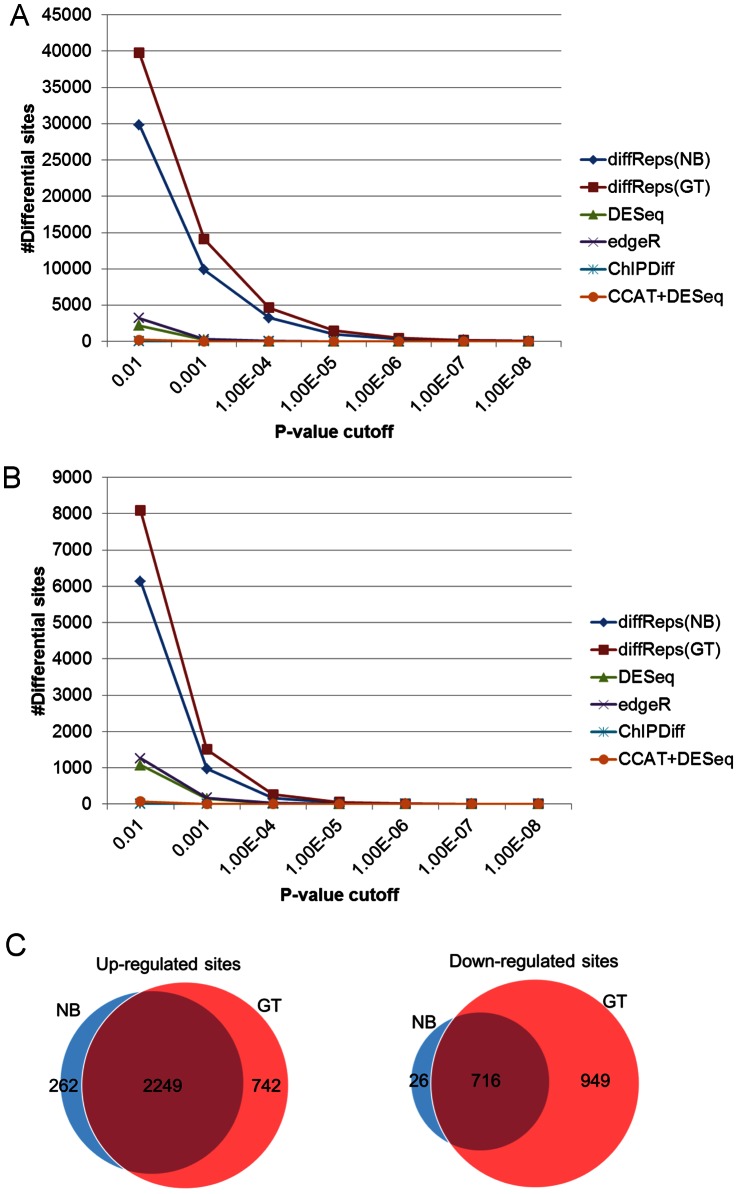
The number of differential sites found by different methods as p-value cutoff varies from 1E-2 to 1E-8 on the brain H3K9me3 ChIP-seq data. NB = negative binomial test. GT = G-test. X-axis represents the different p-value cutoffs. Y-axis represents the number of differential sites. (A) Sensitivity curves based on the H3K9me3 ChIP data; (B) Specificity curves based on the DNA input mock data; (C) Venn diagrams of NB vs. GT using diffReps based on its default cutoff (*p<1E-4*).

First, we performed the same sensitivity and specificity study as above on these data. The results (Fig. 7A; See Figs. S5 & S6 for additional p-values) again show that diffReps is more sensitive than the other methods. Because the “–nsd" option is set to a more permissive threshold than above, we observed more false positives from almost all methods (ChIPDiff is still zero) on the mock data (Fig. 7B). At the most relaxed cutoffs, i.e. *p<1E-2* and *p<1E-3*, the FDR seems to be very high: roughly 10–20% for both negative binomial test and G-test using diffReps. At the default cutoff (*p<1E-4*), the FDR decreases to 5% for negative binomial test and 6% for G-test. Similarly, the negative binomial test result is a subset (nearly) of the G-test result (Fig. 7C).

**Figure 8:**
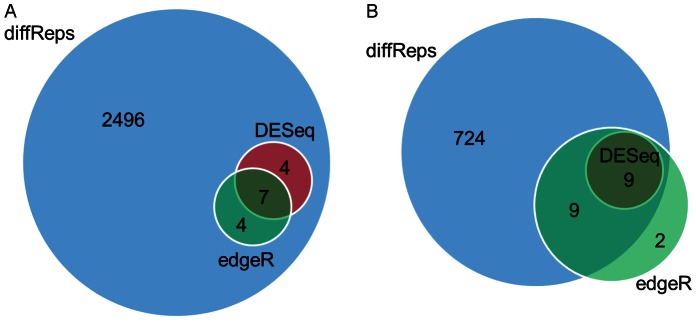
Venn diagrams of the differential sites of diffReps, DESeq and edgeR on H3K9me3 comparing cocaine- and saline-treated mouse nucleus accumbens. The priority for number reporting is set to be: edgeR>DESeq>diffReps. (A) Increased sites; (B) Decreased sites.

**Figure 9:**
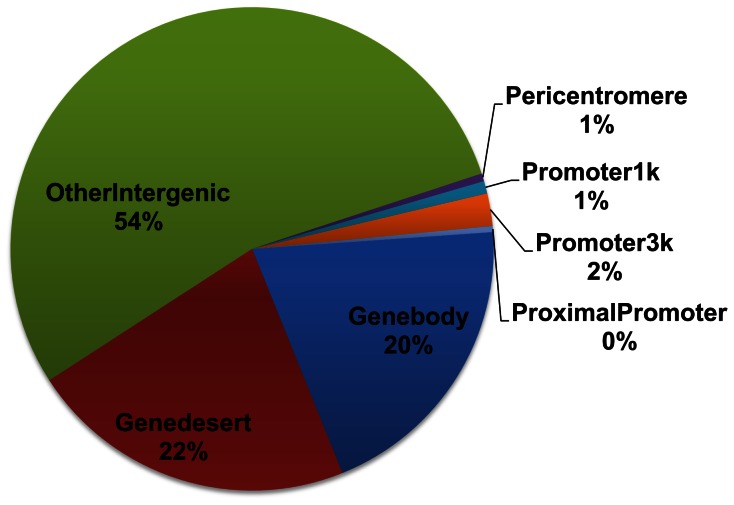
Genomic distribution of the H3K9me3 differential sites identified by diffReps.

We compared diffReps (negative binomial test) with DESeq and edgeR at the default cutoff. diffReps reports more than 3,000 differential sites, while DESeq and edgeR report only 20 and 31 sites. Venn diagrams (Fig. 8) show that diffReps is inclusive of the comparison methods except two down sites that are uniquely identified by edgeR. diffReps automatically annotates the >3,000 differential sites based on their genomic locations. Using the annotated output, we could easily create a pie-chart to analyze the domains of this mark’s regulation. As shown in Fig. 9, the majority of the mark’s regulation resides in intergenic regions (76%). This is consistent with the mark’s enrichment in heterochromatic regions. There is also an appreciable portion of the differential sites in genebodies (20%) with only a very small portion in promoters (3%).

diffReps further defined 266 hotspots (*p<1E-2*; Table S1) from these differential sites. We annotated the hotspots using the ChIPpeakAnno package [18] to identify the overlapping transcripts allowing a 1Kb gap. 132 of the hotspots were found to associate with 627 transcripts which correspond to 198 unique genes. We performed pathway analysis (Ingenuity Systems, www.ingenuity.com) on these genes and found the top one under physiological function to be “Nervous System Development and Function” (*p = 2.9E-8*) which involves 52 genes (Table S2). The first thing we noticed is that the majority of these genes would be considered to be silenced in adult neurons. For example, the top functional annotation is “olfactory response of organism” (*p = 2.9E-8*) that involves 27 olfactory receptors (OR), which are not expressed appreciably outside of the olfactory epithelium; the top two functional annotation are “abnormal morphology of axis” (*p = 5.1E-07*) that involves five homeobox domain genes, which are developmentally regulated transcription factors that function during mammalian development [19]. Our RNA-seq data confirm that these genes are not expressed at significant levels in the nucleus accumbens (unpublished data). Although it is hard to predict what partial unsilencing, or further silencing, of these genes would do in this brain region, regulation at these sites indicates that repeated cocaine might alter certain developmentally determined gene programs, which, through chromatin structural alterations, may lead to aberrant regulation of silenced loci in adult nucleus accumbens neurons. This observation is consistent with our previous study [8] where we also observed global alterations in chromatin/nuclear structure, whereby chronic cocaine exposure resulted in decreased heterochromatic domains and increased nuclear surface area in nucleus accumbens neurons, potentially indicating large scale alterations in nuclear function. What is more, a recent study has shown that OR are being dynamically regulated by H3K9me3, where the choice of expression is mediated via derepression of this histone mark [20]. Our results may then shed light on novel functional roles for this group of genes in cocaine-regulated transcriptional perturbation in nucleus accumbens. In the following, we briefly discuss some other genes that we found to be potentially interesting:

- SYNE1 is a multi-isomeric modular protein that forms a network between organelles and the actin cytoskeleton to maintain the subcellular spatial organization [21]. This could also be important for synaptic development, although there is no literature to indicate a role in cocaine responsiveness.
- HDAC2 has been implicated in dendritic/synaptic regulation in adult brain, both in the context of learning and memory and stress behaviors [22]. Also, it has been observed to increase in expression in response to cocaine self-administration [23].
- RASGRF1 could also be interesting, as Ras signaling (which is induced by increased BDNF signaling in nucleus accumbens) has been shown to be important for cocaine behaviors [24]. RASGRF1 is induced by cocaine, which promotes Ras-Mapk signaling in the nucleus accumbens [25] and is important for phospho-CREB signaling [26] and ΔFosB accumulation [27], both of which are important for cocaine behavior.

In summary, the hotspot-detecting program allows us to identify the chromatin regions that are being heavily regulated. Applying this method to ChIP-seq analysis of H3K9me3 from a drug administration study in mouse brain reveals cocaine-mediated regulation on several genes which may turn out to be novel targets in the future. Therefore, the hotspot-detecting program can be a useful research tool for those who are interested in dynamic regulation of chromatin modifications.

## Materials and Methods

### Algorithms and Workflow of diffReps

The brief workflow of diffReps has been described above. diffReps accepts BED (http://genome.ucsc.edu/FAQ/FAQformat#format1) files as input. BED is a simple text format that can be conveniently converted from any alignment format. For convenience, the diffReps package includes two scripts to convert from SAM [6] or Illumina’s ELAND export/sorted formats to BED. We have also allowed a user to set the average fragment size (–frag) in his/her ChIP-seq libraries. This argument will be used to shift the position of each short read towards the 3′ end by half of the average fragment size. Because the short reads are measured from both ends of the sequenced DNA fragments, this process can improve the accuracy of read count for each sliding window. This design is similar to the ones that have been implemented in other peak calling programs such as MACS [28].

The reference genome typically contains large blocks of unmappable regions such as repetitive regions. In addition, the majority of the genome often does not show significant ChIP enrichment. To increase statistical power and save computational cost, diffReps performs a prescreening of the genome and filters those regions with low read count. To set a reasonable cutoff, diffReps estimates the background mean and deviation using robust statistics, which are implemented by right-trimmed mean (controlled by option –alpha) and median absolute deviation (MAD, with center = right-trimmed mean). This is motivated by the observation that certain genomic regions may contain abnormally high read counts. High read counts can be caused by a few factors, such as uneven chromatin structures or biased PCR amplification. Therefore, using robust statistics can prevent them from distorting the background estimation. In addition, we randomly sample 100,000 non-overlapping windows without replacement from the genome to calculate the above statistics. Sliding windows with read counts smaller than this formula are then filtered:
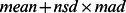
where “nsd” is a tunable integer parameter (option –nsd). Two default modes have been defined for histone marks with sharp (nsd = 20) and broad (nsd = 2) peaks.

Normalization is accomplished by calculating a numeric factor for each sample so that each raw read count can be linearly scaled using its corresponding factor. Assume all samples and windows are identified by subscript *i* and *j*, respectively, we calculate these factors by two steps: 1. At each window *j*, we calculate a factor *f_ij_* for each sample *i* using the following formula:
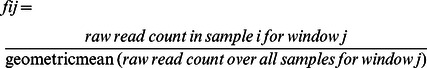



2. We use the median of all *f_ij_* (which are larger than zero) over subscript *j* as a representative factor for each sample *i*. Here, median is used instead of mean to avoid outliers. By default, the normalization is only done on the windows that pass the aforementioned low count cutoff. This default behavior can be overridden by using all windows on the genome (option *–nrpass*).

When there are biological replicates in a dataset, negative binomial test is the recommended approach for differential analysis. We have implemented an exact negative binomial test in diffReps, which follows that of Anders and Huber [10]. Let’s use subscript “*tre*” and “*con*” to denote the treatment and control groups. Given summed raw read counts *k_tre_* and *k_con_* from the treatment and control groups, and *k_tre_*+*k_con_* = *k_total_*, the p-value of (*k_tre_*, *k_con_*) is the summation of the probabilities of all pairs with probabilities less than or equal to *p*(*k_tre_*, *k_con_*) among all combinations, i.e.
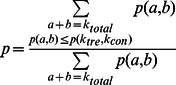
where 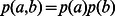 assuming the two groups are independent; 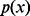 is computed using a negative binomial distribution.

Because negative binomial distribution models discrete count data and over-dispersion among different replicates, it appears to be an ideal model for ChIP-seq data. However, many studies to date have used T-tests on normalized counts for differential analysis. This is sub-optimal because normalized counts are not normally distributed. As a result, detection power can be significantly degraded. Another caveat about T-tests is that regions with very small counts may be picked up. Those regions should never pass cutoff because small counts are associated with very little confidence and they are very likely to be background noises. T-tests simply ignore this fact because they treat normalized counts as continuous values. Di et al. [29] have evaluated negative binomial test, Poisson test and T-test on RNA-seq data and found the negative binomial test to be vastly superior. We provide T-tests in diffReps package mainly for comparison purposes.

If an experiment does not contain biological replicates, users can choose between G-test and *χ^2^* test for differential analysis. The two tests give similar results but G-test is more preferred and has gained in popularity recently [30]. When these two tests are chosen, diffReps performs a goodness-of-fit test on the normalized counts of the treatment and control groups. G-test or *χ^2^* test can also be used on data with biological replicates. An incentive of doing this is that it may increase sensitivity though it can incur more false positives. When this approach is being used, diffReps automatically combines the biological replicates and generates a probability vector accordingly. Assume the summation of the normalized counts for the treatment and control group is *o_tre_* and *o_con_*, respectively; the number of replicates is *r_tre_* and *r_con_*, respectively. We first calculate a probability vector as
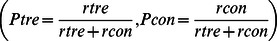



Then we need to calculate the expected counts for the treatment and control group as
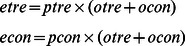



If *χ^2^* test is being used, we calculate the statistic as
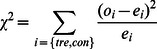



If G-test is being used, we calculate the statistic as
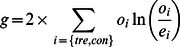



The significance for the above two statistics is evaluated by
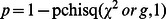
Where *pchisq* is the cumulative distribution function of a *χ^2^* distribution and here the degree of freedom is always 1 (because we only have two groups).

diffReps performs statistical tests on sliding windows and the significant windows are selected by a predefined cutoff (option *–pval*). The significant windows that overlap with each other are then merged and the differential sites are used to perform the statistical tests again. Finally, the *p-value* for each differential site and the best *p-value* for the sliding window within each differential site are reported. *P-values* are adjusted by the Benjamini-Hochberg (BH) method [31].

After the differential sites have been found, diffReps assigns each of them to a nearby gene. This is implemented through a “refgene_getnearestgene” program from the CisGenome package [10]. If no gene is assigned, diffReps tries to associate the differential sites with a heterochromatic region [32] through the “intersectBed” program from BEDTools package [33]. diffReps then attaches these annotation information to its output for further analysis.

### Hotspot Detection

To detect hotspots of chromatin modifications, all differential sites are first sorted according to their genomic coordinates in ascending order and stored in a list as: *d_1_,d_2_,…,d_N_* where *N* is the total number of differential sites. Any two differential sites, *d_i_* and *d_j_* (*i<j*), along with all the differential sites in between, form a so called “site stretch”. The number of segments of a stretch, defined as *j-i*, can be approximated by a Poisson distribution if all differential sites were positioned on the genome by random allocation. diffReps finds the hotspots by three steps:

1. Look for a stretch that is most likely to be a hotspot by greedy search;
2. Build a local Poisson model surround the stretch;
3. Test for its statistical significance.

One may think the number of differential sites per hotspot should be used to define the “tightness” of the hotspot. However, it becomes ambiguous to assign the sites on the boundaries to hotspot or background. To avoid this ambiguity, we use the number of segments inside a hotspot to define its tightness. This basically equals cutting each site on the boundary into two halves.

diffReps searches for hotspots on a chromosome from left to right using a greedy search strategy. If the *p-value* for a candidate hotspot is significant, diffReps keeps it in memory and continues to add another differential site on the right to see if the *p-value* can be improved. Sometimes, this process may reach a local optimum when a previously added site is more distant to the rest of the hotspot than the most recently added site. To avoid this trap, diffReps attempts to remove the left most site and test the *p-value* again each time a new site is added. If the *p-value* is decreased, this procedure can repeatedly remove the left most site so that we are always getting a “tightest” hotspot. This algorithm is summarized in the following:

1. Initialize a hotspot by the first differential site and set p-value to 1.0;
2. Repeat the following procedure until all sites are exhausted:
  1. Add a site to the right and calculate a p-value;
  2. Compare this p-value with the previous p-value:

if current p-value <previous p-value {.

Keep the new site;

Remove the left most site to see if the score can be improved;

if yes {.

Keep the change and repeatedly remove site from the left until no improvement can be achieved.

} else {.

Add the left most site back into the candidate hotspot.

}.

} else {.

Pop the newly added site;

Output the current hotspot if it passes significance cutoff;

Initialize a new hotspot with the current site.

}.

1. Adjust p-values in the list and output all hotspots.

diffReps uses local Poisson model to describe the number of segments in a stretch. This is achieved by first estimating the number of segments per nucleotide in a genomic region of 200Kb or 1Mb on each side of the site stretch, denoted as *λ_200Kb_* and *λ_1Mb_*. diffReps also calculates a global value at the beginning using all the differential sites on all chromosomes combined as a site stretch, which is denoted as *λ_global_*. The maximum of the three estimates is used as
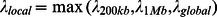



diffReps then calculates the number of expected segments in the stretch by
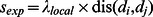
where *dis* is a function to calculate the number of nucleotides from *d_i_*’s center to *d_j_*’s center and always yields an infinite value if *d_i_* and *d_j_* are located on two different chromosomes. The statistical significance for the tightness of the site stretch is evaluated by



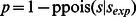
where *s = j-i* is the number of segments observed in the stretch and *ppois* gives the cumulative distribution function of a Poisson distribution. A predefined cutoff can be set at the beginning to identify candidate hotspots. Upon completion, *p-values* are adjusted by the BH method [31].

Upon completion, diffReps reports all the information about a hotspot, such as its genomic coordinates, *p-value*, histone mark types and the original record locations. In addition, diffReps can accept more than one list of different histone marks as input. In this case, diffReps merges all differential lists and sorts differential sites in ascending order before searching. Users may use the histone mark types in all hotspots to predict the interaction between two or more histone marks.

### Installation

Installing diffReps is just like a standard PERL module. Basically users extract the package downloaded, go to the program directory and type the following commands:

perl Makefile.PL (Optional, PREFIX = your_perl_directory).

make.

make test.

make install.

If a user has root privileges, diffReps.pl will most likely be installed in/usr/bin/. If user specified PREFIX in Makefile, it will be installed in user_perl_directory/. Add user_perl_directory/bin to user’s PATH environmental variable, or copy diffReps.pl from user_perl_directory/bin to a directory that is already in PATH, such as/home/user_name/bin.

Alternative: If user has cpanminus installed, user can also install diffReps with one line command:

cpanm diffReps-XXX.tar.gz.

it will try to satisfy all the dependencies for the user.

### Availability and Future Directions

diffReps packages are available at https://code.google.com/p/diffreps/under GPL v3 license. Future improvements include support for BAM files, multi-threading for computationally intensive routines and handling multi-reads.

## Supporting Information

Figure S1 **Overlap of the top differential site lists of three methods: diffReps (negative binomial test), edgeR and CCAT+DESeq on H3K4me3 comparing K562 and hESC.** (A) Top 2,500; (B) Top 5,000.
(PDF)

Figure S2 **Distance density plot of all diffReps-specific sites (10,025 in total with 3 on chrM excluded).** The distance is determined by first looking for the closest TSS or alternative exon and then calculating the number of basepairs between the boundaries of a site and a feature. 9,168 sites are assigned to TSS, of which 7,783 are within 1Kb. 857 sites are assigned to exon, of which 627 are within 1Kb. The density plots are cut at 1Kb window size. (A) Distance to TSS; (B) Distance to exon.
(PDF)

Figure S3 **The number of differential sites found by different methods as p-value cutoff varies from 0.5 to 0.1 on the ENCODE H3K4me3 ChIP-seq data.** NB = negative binomial test. GT = G-test. X-axis represents the different p-value cutoffs. Y-axis represents the number of differential sites. (A) Sensitivity curves based on the H3K4me3 ChIP data; (B) Specificity curves based on the DNA input mock data.
(TIF)

Figure S4 **Total size of the genomic regions that are covered by differential sites from different methods as p-value cutoff varies from 0.5 to 1E-8 on the ENCODE H3K4me3 ChIP-seq data.** NB = negative binomial test. GT = G-test. X-axis represents the different p-value cutoffs. Y-axis represents the genomic region size in Mb. (A) Sensitivity curves based on the H3K4me3 ChIP data; (B) Specificity curves based on the DNA input mock data.
(TIF)

Figure S5 **The number of differential sites found by different methods as p-value cutoff varies from 0.5 to 0.1 on the brain H3K9me3 ChIP-seq data. NB = negative binomial test. GT = G-test.** X-axis represents the different p-value cutoffs. Y-axis represents the number of differential sites. (A) Sensitivity curves based on the H3K9me3 ChIP data; (B) Specificity curves based on the DNA input mock data.
(TIF)

Figure S6 **Total size of the genomic regions that are covered by differential sites from different methods as p-value cutoff varies from 0.5 to 1E-8 on the brain H3K9me3 ChIP-seq data.** NB = negative binomial test. GT = G-test. X-axis represents the different p-value cutoffs. Y-axis represents the genomic region size in Mb. (A) Sensitivity curves based on the H3K9me3 ChIP data; (B) Specificity curves based on the DNA input mock data.
(TIF)

Figure S7 **An additional example of an H3K4me3 differential site (diffReps-specific) that may associate with splicing.** The top two tracks are normalized genomic coverage of H3K4me3 in K562 and hESC cell lines. They are overlaid by diffReps-specific sites shown as solid bars. The bottom track is the gene model with two representative isoforms. Gene ASUN contains alternative splicing with a variant exon being preferentially excluded in K562 vs. hESC. *SI = *−0.1 for ENST00000261191 and *SI* = 0.1 for ENST00000539625.
(PNG)

Table S1 **Hotspot list identified from brain H3K9me3 ChIP-seq data. P-value <0.01 is used as a cutoff.**
(XLSX)

Table S2 **Gene ontology terms enriched in the H3K9me3 hotspots.** The top one enriched category is “Nervous System Development and Function” which involves 52 genes. Here is a list of all the sub-terms under this category.
(XLSX)

Table S3 **Numbers of overlapped genes between transcriptional changes and differential H3K4me3 sites.** These numbers are used to derive the statistical significance in Fig. 4 using Fisher’s exact test.
(XLSX)

Text S1 **Supplemental materials and analysis.**
(DOC)
