# Supplementary figures and images for: diffReps: Detecting Differential Chromatin Modification Sites from ChIP-seq Data with Biological Replicates
Source: PLoS One. 2013 Jun 10;8(6):e65598. doi: 10.1371/journal.pone.0065598 (PMC3677880; doi:10.1371/journal.pone.0065598)

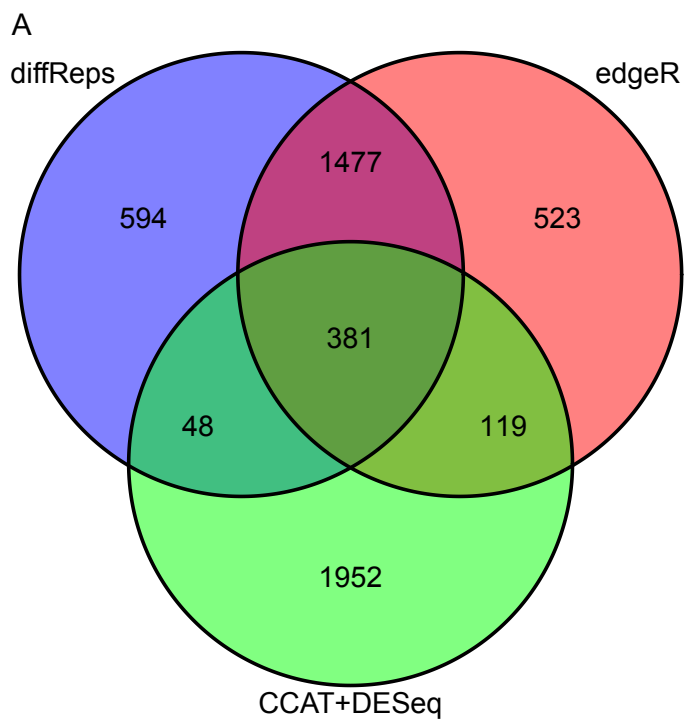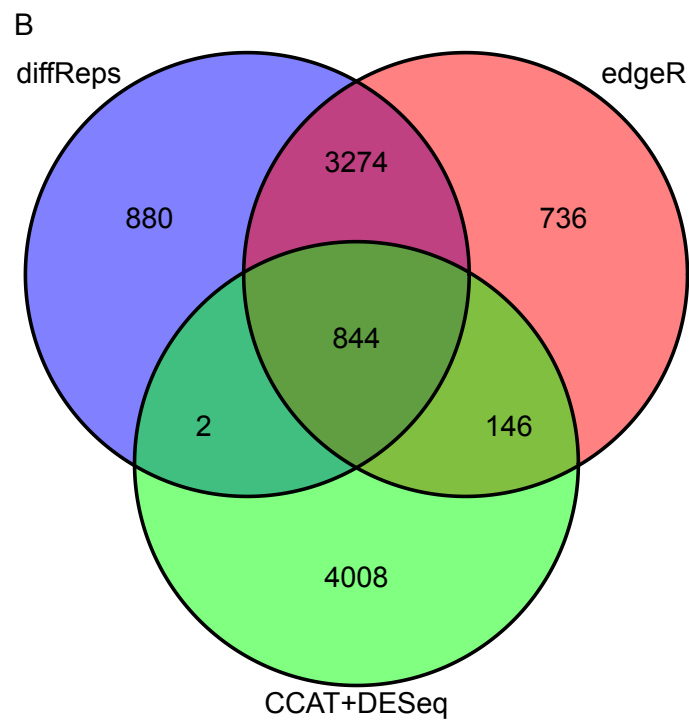

Supplement: Figure S1 — Overlap of the top differential site lists of three methods: diffReps (negative binomial test), edgeR and CCAT+DESeq on H3K4me3 comparing K562 and hESC. (A) Top 2,500; (B) Top 5,000. (PDF) [file pone.0065598.s001.pdf]

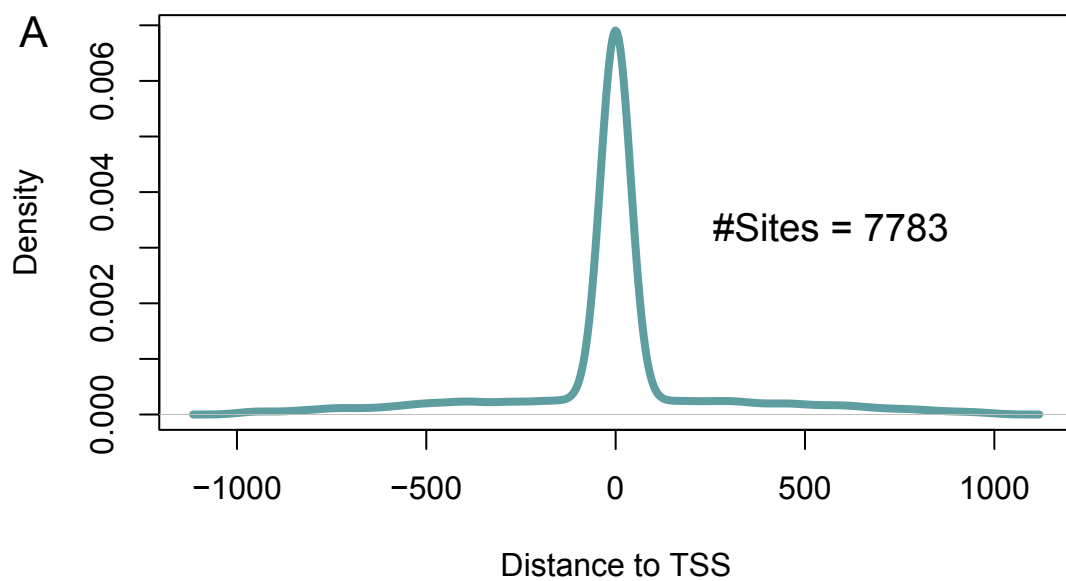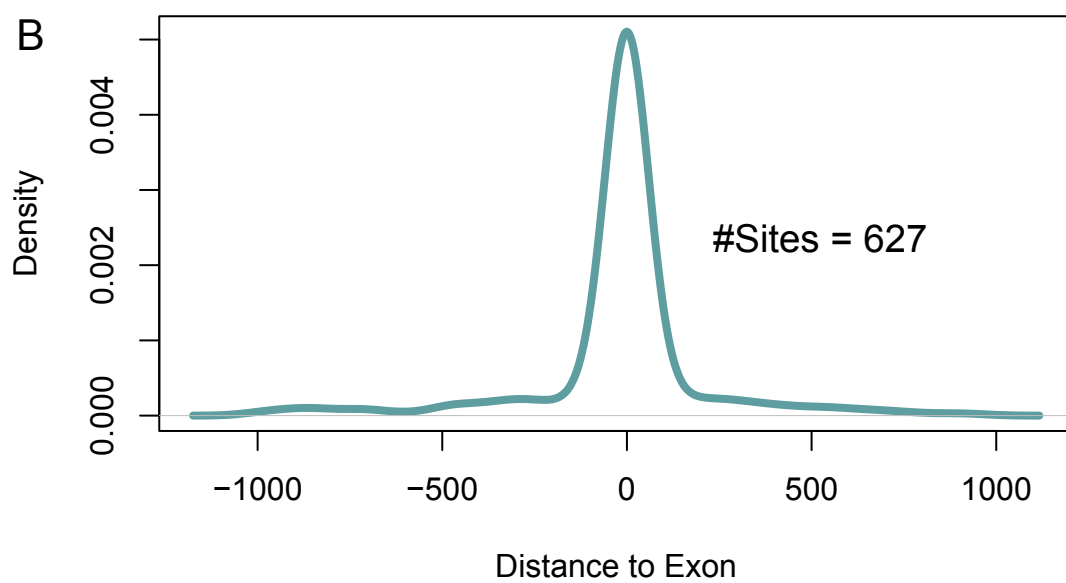

Supplement: Figure S2 — Distance density plot of all diffReps-specific sites (10,025 in total with 3 on chrM excluded). The distance is determined by first looking for the closest TSS or alternative exon and then calculating the number of basepairs between the boundaries of a site and a feature. 9,168 sites are assigned to TSS, of which 7,783 are within 1Kb. 857 sites are assigned to exon, of which 627 are within 1Kb. The density plots are cut at 1Kb window size. (A) Distance to TSS; (B) Distance to exon. (PDF) [file pone.0065598.s002.pdf]

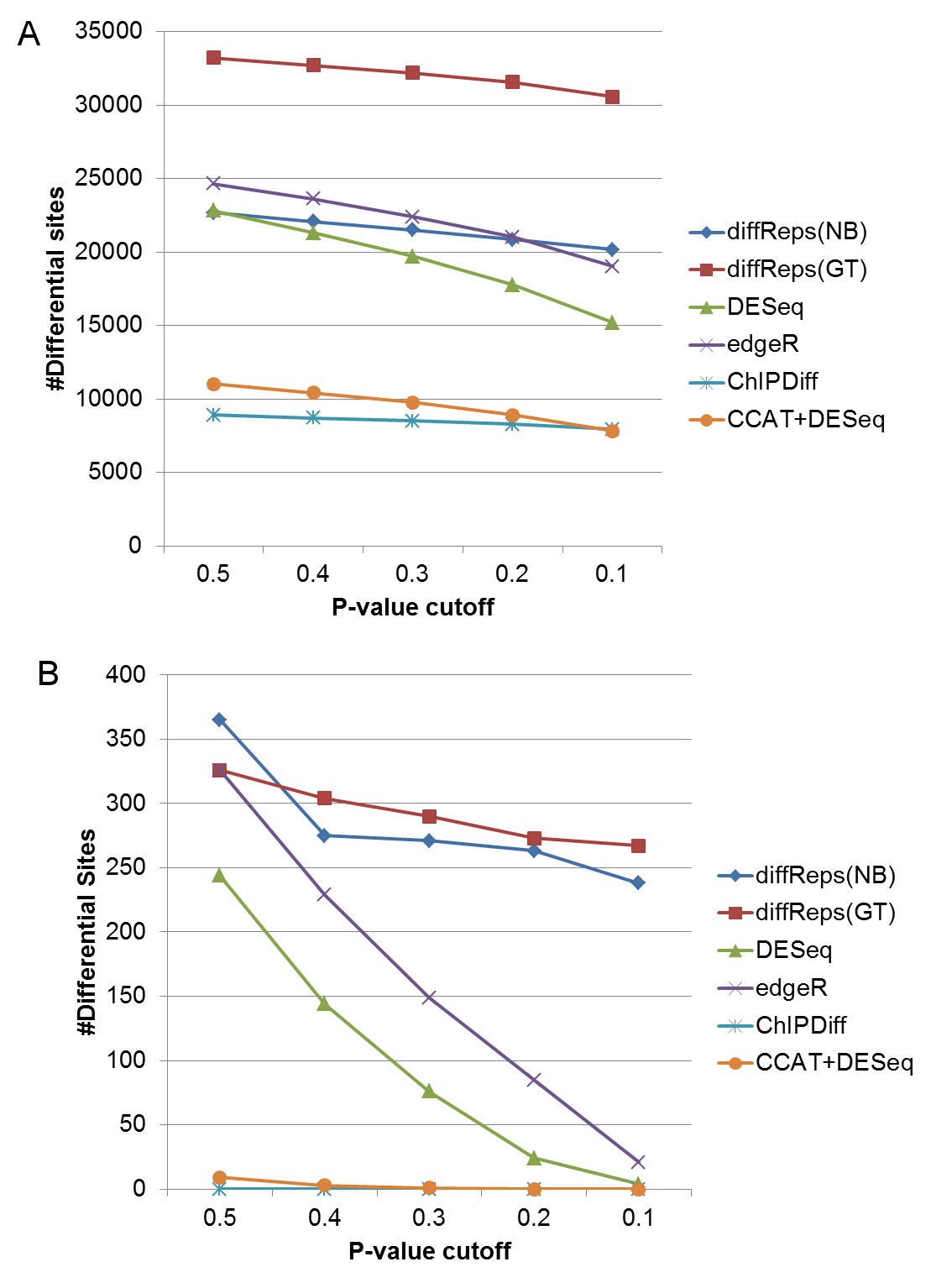

Supplement: Figure S3 — The number of differential sites found by different methods as p-value cutoff varies from 0.5 to 0.1 on the ENCODE H3K4me3 ChIP-seq data. NB = negative binomial test. GT = G-test. X-axis represents the different p-value cutoffs. Y-axis represents the number of differential sites. (A) Sensitivity curves based on the H3K4me3 ChIP data; (B) Specificity curves based on the DNA input mock data. (TIF) [file pone.0065598.s003.tif]

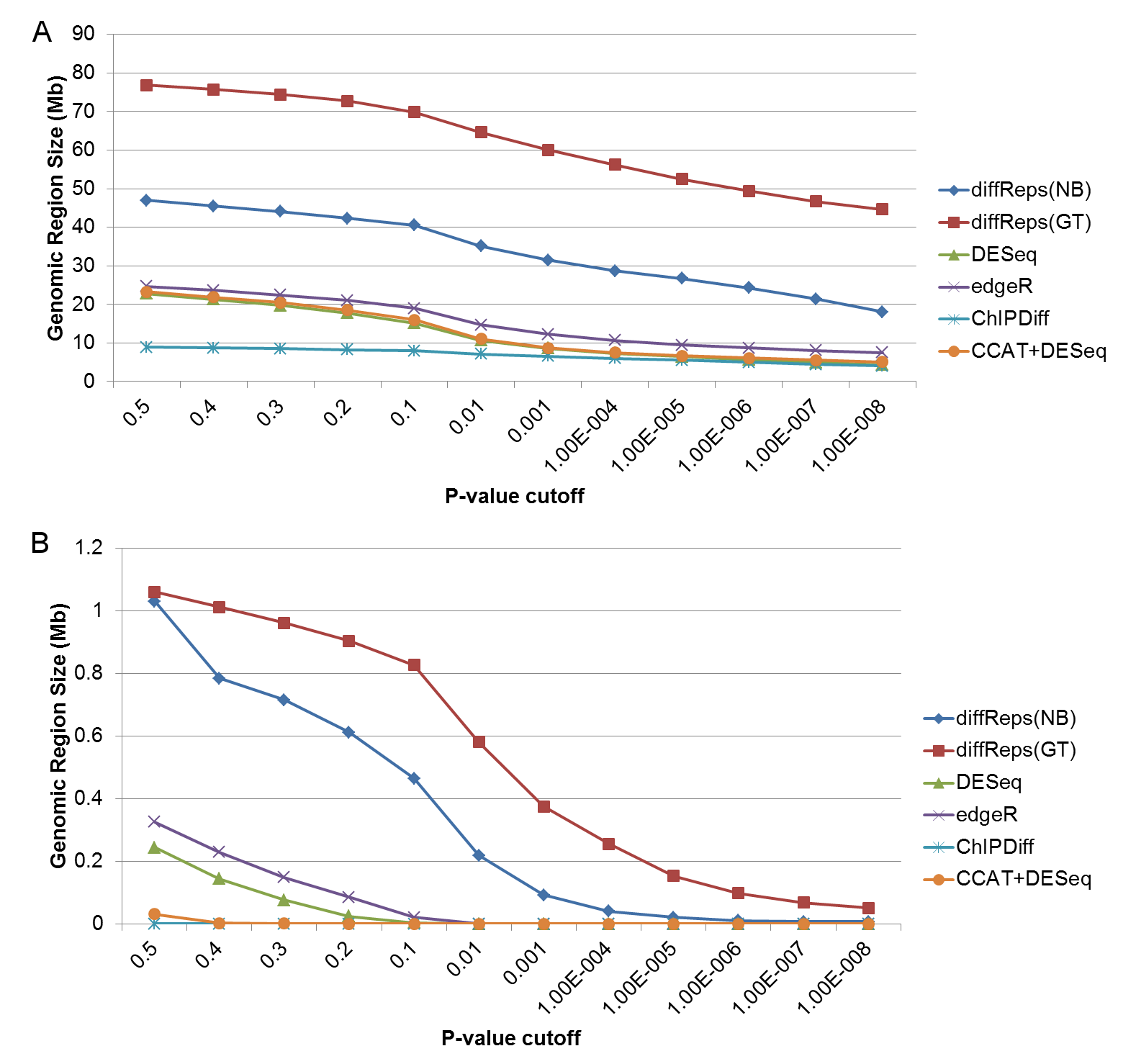

Supplement: Figure S4 — Total size of the genomic regions that are covered by differential sites from different methods as p-value cutoff varies from 0.5 to 1E-8 on the ENCODE H3K4me3 ChIP-seq data. NB = negative binomial test. GT = G-test. X-axis represents the different p-value cutoffs. Y-axis represents the genomic region size in Mb. (A) Sensitivity curves based on the H3K4me3 ChIP data; (B) Specificity curves based on the DNA input mock data. (TIF) [file pone.0065598.s004.tif]

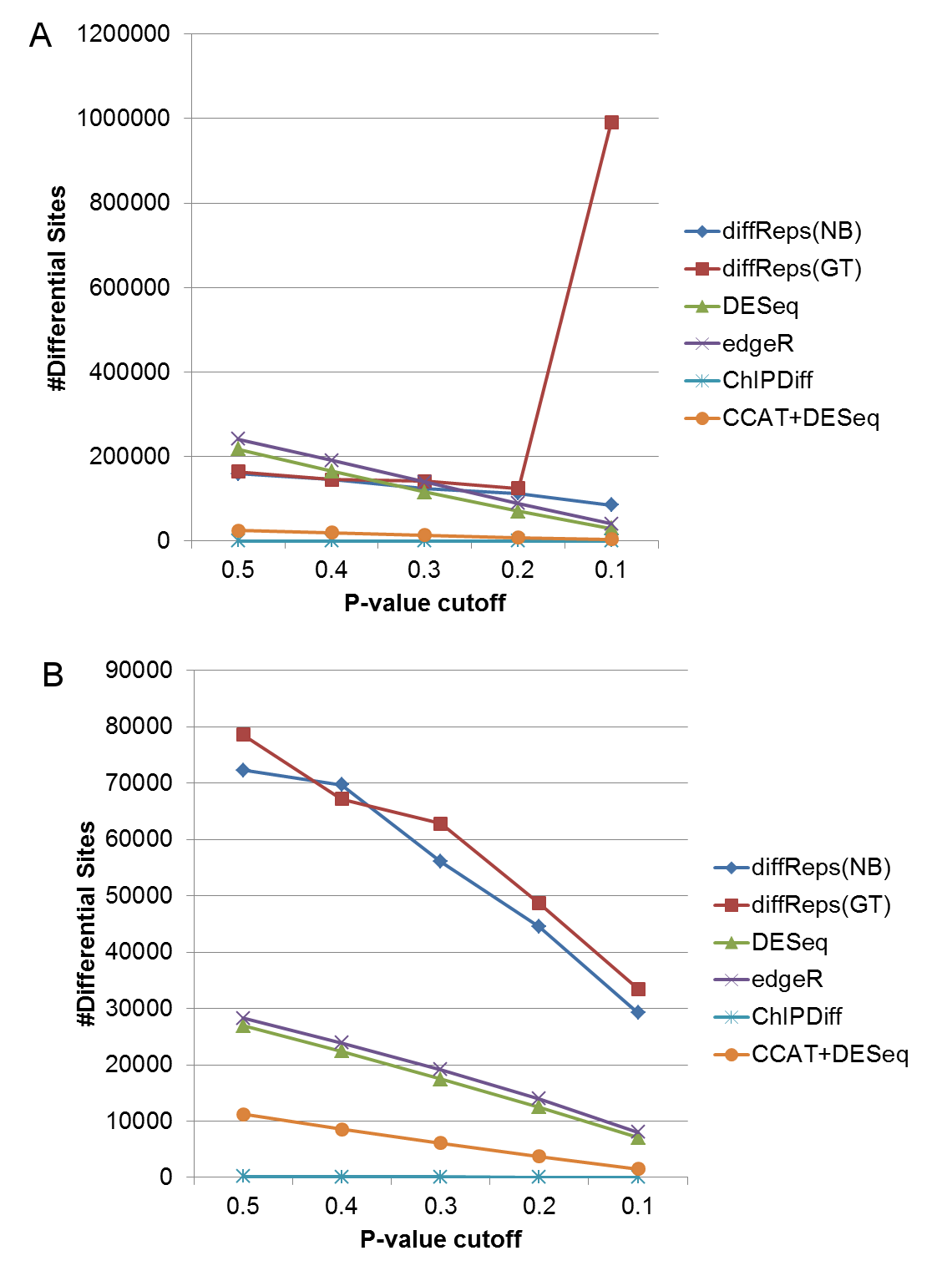

Supplement: Figure S5 — The number of differential sites found by different methods as p-value cutoff varies from 0.5 to 0.1 on the brain H3K9me3 ChIP-seq data. NB = negative binomial test. GT = G-test. X-axis represents the different p-value cutoffs. Y-axis represents the number of differential sites. (A) Sensitivity curves based on the H3K9me3 ChIP data; (B) Specificity curves based on the DNA input mock data. (TIF) [file pone.0065598.s005.tif]

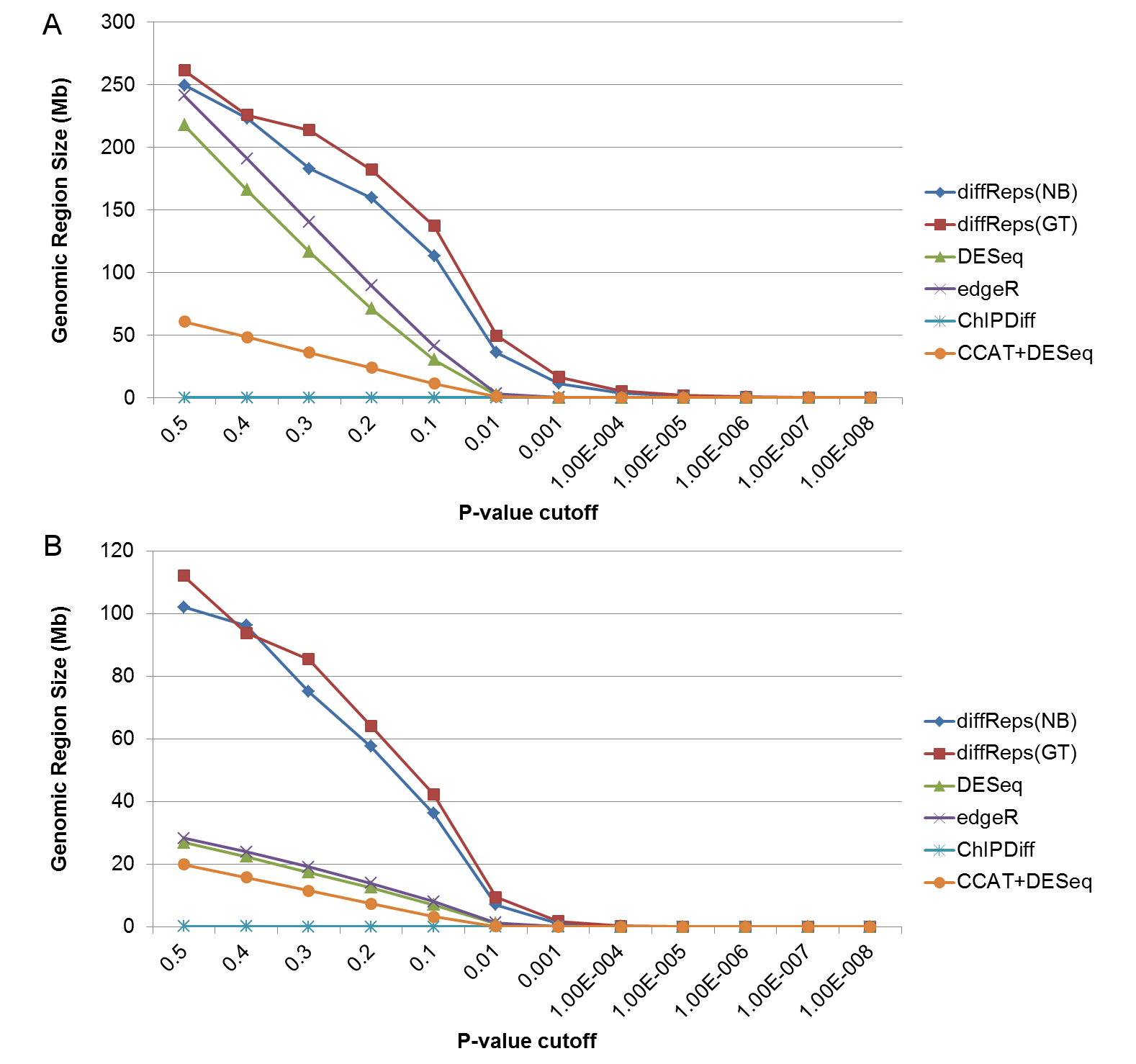

Supplement: Figure S6 — Total size of the genomic regions that are covered by differential sites from different methods as p-value cutoff varies from 0.5 to 1E-8 on the brain H3K9me3 ChIP-seq data. NB = negative binomial test. GT = G-test. X-axis represents the different p-value cutoffs. Y-axis represents the genomic region size in Mb. (A) Sensitivity curves based on the H3K9me3 ChIP data; (B) Specificity curves based on the DNA input mock data. (TIF) [file pone.0065598.s006.tif]

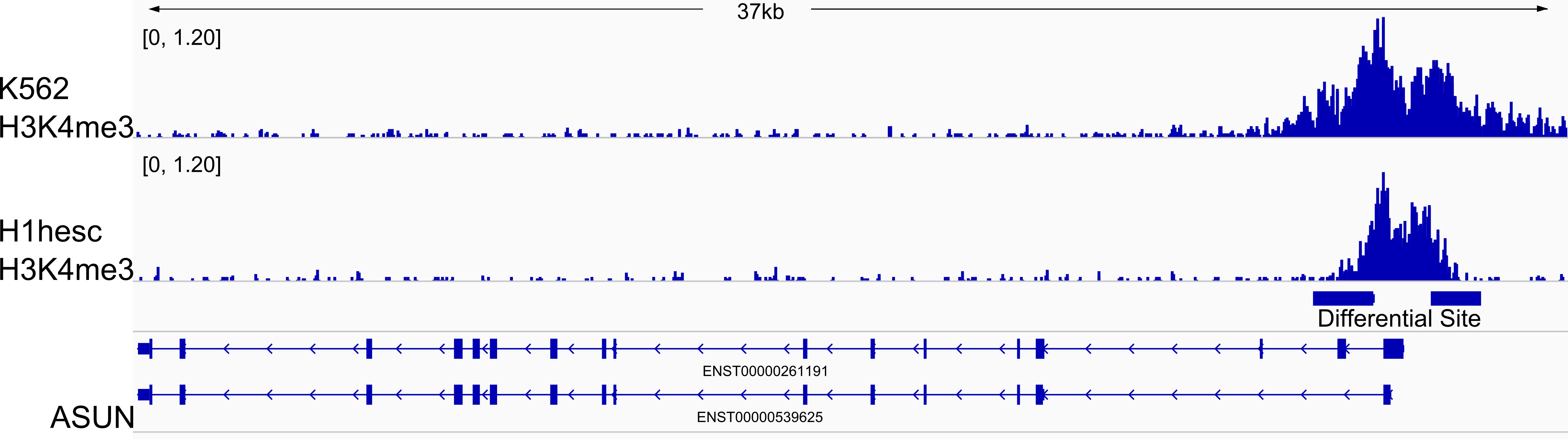

Supplement: Figure S7 — An additional example of an H3K4me3 differential site (diffReps-specific) that may associate with splicing. The top two tracks are normalized genomic coverage of H3K4me3 in K562 and hESC cell lines. They are overlaid by diffReps-specific sites shown as solid bars. The bottom track is the gene model with two representative isoforms. Gene ASUN contains alternative splicing with a variant exon being preferentially excluded in K562 vs. hESC. SI = −0.1 for ENST00000261191 and SI = 0.1 for ENST00000539625. (PNG) [file pone.0065598.s007.png]
