# Supplementary material for: diffReps: Detecting Differential Chromatin Modification Sites from ChIP-seq Data with Biological Replicates
Source: PLoS One. 2013 Jun 10;8(6):e65598. doi: 10.1371/journal.pone.0065598 (PMC3677880; doi:10.1371/journal.pone.0065598)
Supplement: Text S1 — Supplemental materials and analysis. (DOC) [file pone.0065598.s011.doc]

# Supporting Information

## Supplemental Materials and Analysis

### Details of the ENCODE Dataset

The human cell lines and their corresponding data can be found at: <http://genome.ucsc.edu/ENCODE/dataMatrix/encodeDataMatrixHuman.html>. The Gene Expression Omnibus (GEO) sample accession numbers of H3K4me3 are GSM733657 (H1 hESC), and GSM733680 (K562). The accession numbers of input are GSM733770 (H1 hESC), and GSM733780/GSM822293 (K562) . There are two replicates for every cell line.

Gene expression analysis was based on RNA-seq data and the accession numbers are GSM758566 (H1 hESC) and GSM765405 (K562). The RNA-seq data are paired-end and have two replicates per cell line.

### Details of the *in vivo* brain dataset

This dataset is available using the GEO accession number: GSE24850

### Comparing diffReps and other methods

To ensure fair comparisons, we used the genomic regions that passed the same “--nsd" cutoff as diffReps to filter the results generated by all methods. For DESeq and edgeR, we first split the whole genome into non-overlapping windows at 1Kb size and then performed differential analysis using their default parameter values. We did not attempt to merge the adjacent windows that are identified as significant.

For ChIPDiff and CCAT, the replicates in each group were pooled because they do not support the use of replicates. We then applied them with the window size of 1Kb. After CCAT found peaks in control and treatment groups, the union of the two peak lists was used to calculate read count for each replicate. These read count information were then given to DESeq to identify differential sites.

ChIPDiff works by modeling the log fold change of each non-overlapping window and merges the adjacent windows that are classified as significant. The merged windows are used to define a differential site. However, ChIPDiff does not provide additional information about the differential sites besides their genomic coordinates. We were not able to rank those differential sites. Therefore, we decided to report the number of non-overlapping windows instead of the number of differential sites. As a result, the number for ChIPDiff reported in this paper is always larger than the program’s default number. The non-overlapping windows of ChIPDiff were ranked according to fold changes in descending order.

### Additional p-values for the sensitivity and specificity curves

Additional p-values: 0.5, 0.4, 0.3, 0.2 and 0.1 are added in Figs. S3 & S5. Basically, the curves in this range follow similar trends as p-value from 1e-2 to 1e-8. However, the curves are not necessarily monotonic for diffReps at large p-values; at certain p-values, diffReps reports less differential sites than DESeq and edgeR. This is because diffReps merges a lot of sliding windows into one differential site if they overlap. To show the sensitivity from another angle, we also illustrate the total size of the genomic regions that are covered by the differential sites in Figs. S4 & S6. In these comparisons, diffReps is consistently more sensitive than the other methods at all p-values.

### Computational cost and memory usage

We recorded the running time and memory usage of diffReps for the two datasets used in this manuscript on a single core node with CPU speed of 2.1GHz. On the *in vivo* mouse brain data, diffReps finished running in 1h20m with maximum memory usage of 3.2GB; on the ENCODE data, diffReps finished running in 2h30m with maximum memory usage of 2.8GB.

Some analyses for the running time and memory usage are as follows:

- The preparation of bin count data scales linearly with data size (i.e. the number of short reads);
- The bin scanning and testing time scales linearly with the number of bins which equals genome size times the inverse of window size;
- Memory usage scales linearly with bin number times the number of replicates.

The results conclude that diffReps can work on a typical real-world dataset of mammalian genome size with reasonable running time and memory requirement.

## References

1. (2011) A user's guide to the encyclopedia of DNA elements (ENCODE). PLoS biology 9: e1001046.
